# Differences in Postural Control Associated With Aging and Executive Function

**DOI:** 10.7759/cureus.81771

**Published:** 2025-04-05

**Authors:** Kensuke Matsuda, Issei Murai, Ryota Okoba, Takuro Ikeda, Yoshio Takano

**Affiliations:** 1 Department of Physical Therapy, School of Health Sciences at Fukuoka, International University of Health and Welfare, Okawa, JPN; 2 Department of Rehabilitation, Takaki Hospital, Okawa, JPN; 3 Department of Physical Therapy, Faculty of Health and Welfare, Prefectural University of Hiroshima, Mihara, JPN; 4 Faculty of Medical Sciences, Fukuoka International University of Health and Welfare, Fukuoka, JPN

**Keywords:** adults, aging, cognitive function, executive function, falls risk, postural control

## Abstract

Objective: Standing postural instability is associated with age-related functional decline and risk of fall. This study investigated the ability to control posture during stationary standing in young adults, older adults, and at-risk older adults from the perspective of postural sway and cumulative lower limb angles.

Methods: The participants were instructed to assume a stationary upright position in three conditions: unstable, stable, and unstable with executive function tasks. Postural control was quantitatively assessed using a center-of-gravity sway meter and angular velocity sensors.

Results: The 58 participants (all female participants) included in this study were divided into groups of young adults (n = 21), older adults (n = 25), and at-risk older adults (n = 12). Compared with younger adults, older adults and at-risk older adults showed decreased executive function and increased total pressure swing length under unstable conditions. The cumulative angles of the ankle joints also increased. Similar results were observed under unstable and attention-dispersal conditions. Additionally, older adults and at-risk older adults showed increased cumulative knee and hip angles.

Conclusion: Older adults and at-risk older adults experience postural instability and use different postural control strategies compared with younger individuals under unstable or unstable conditions with attention dispersion.

## Introduction

Falls are a major global health concern, contributing to fractures and increased medical costs. Approximately 40% of ambulatory older adults aged 70 years and older experience at least one fall per year [1]. The risk of falls increases with declining cognitive function. Falls, which can occur as a consequence of difficulties in maintaining balance, are related to multiple factors [2], including age-related decline in physical function (impaired vision, muscle weakness, etc.) and environmental influences (such as poor lighting and uneven walking surfaces). Balance involves integration and regulation of multiple systems, encompassing several sensory and perceptual processes, cognitive influences, and motor skills. The task of maintaining an upright position on an unstable floor involves cognitive action on multiple domains to process various types of information to maintain posture and effectively adapt to changing environments [3]. Multiple areas are involved in this process, including the prefrontal cortex and dorsolateral prefrontal cortex (executive function and attention), the hippocampus (memory), and the occipital lobes (visual processing and processing speed). These tasks are strongly correlated with executive function, as well as static and dynamic balance [4]. Imaging studies have reported common anatomical and pathological bases for executive and motor functions. A decrease in the volume of the prefrontal and frontoparietal regions, which are involved in executive function, is related to gait disturbances [5]. Aging affects executive function, which in turn affects balance and increases the risk of falls. At present, risk of falls is assessed using single cognitive function tests (e.g., Mini Mental State Examination and Trail Making Test), while secondary tasks requiring executive function are not assessed.

Aging is also associated with postural maintenance and balance problems owing to postural instability. Postural control is important in older adults who are at a high risk of falling and is affected by both physical and cognitive demands [6]. Therefore, allocation and coordination between the motor and cognitive systems are essential. This coordination is dependent on executive function; however, aging limits the cognitive function that assists motor function [7].

Cognitive-motor dual-tasking (CMDT) studies have explored the attention to the motor and cognitive systems involved in postural regulation using dual tasks to determine the extent of performance decline and cognitive recruitment. Complex cognitive loading during dual tasks reduces postural stability and increases gait parameters [8]. Older adults exhibit poorer balance control than young adults under dual-task conditions [9]. However, data on postural control differences between typical healthy older adults and older adults at risk of falls are lacking.

Therefore, this study investigated the ability of young female adults, older female adults, and older female adults at risk of falling to maintain their posture on stable surfaces, unstable surfaces, and unstable surfaces while performing executive function tasks. Postural sway and instability in older adults have been discussed based on observations of the center of pressure (COP) in previous studies on static postural control using dual tasks [10,11]. However, strategies for maintaining postural control while standing and other aspects remain to be clarified. We hypothesized that the cumulative angles of lower extremity joint motion involved in standing postural instability in relation to aging and executive function would differ among young adults, older adults, and at-risk older adults.

## Materials and methods

Ethics statements

The study was approved by the Ethics Committee of the International University of Health and Welfare (approval number: 19-Ifh-081). Written and oral explanations were provided, and informed consent was obtained from all participants.

Participants and setting

This cross-sectional study was conducted at the Department of Physiotherapy, Faculty of Health and Medical Sciences, International University of Health and Welfare, Fukuoka, Japan. Female participants aged 20-22 years were recruited from the university, whereas those aged 65-80 years were recruited from the Silver Human Resource Center. Participants belonging to these age groups were recruited to evaluate the influence of aging on the ability to maintain an upright position and to include older adults at risk of falls. The inclusion criteria were as follows: fulfillment of age requirements, ability to agree verbally and in writing to participate in the study, and ability to walk without support. Conversely, the exclusion criteria were as follows: history of central nervous system or serious orthopedic diseases, visual impairment, and inability to maintain an upright position. A power analysis using G*Power 3.1.9.7 was used to determine the sample size needed to compare the three groups. At an alpha value of 0.05 and power of 0.90, the total calculated sample size was 68.

Protocol design and measurements

The participants' baseline characteristics, including age, body mass index (BMI), skeletal muscle mass index (SMI), functional reach test (FRT) results, executive function, and mobility were recorded after obtaining consent. SMI was measured with bioelectrical impedance analysis (BIA) using a body component analyzer (InBody 270; InBody Japan, Tokyo, Japan). A decrease in skeletal muscle mass is associated with the occurrence of sarcopenia and frailty. The Asian Working Group for Sarcopenia (AWGS) 2019 criteria define reduced skeletal muscle mass as SMI of <5.7 kg/m^2^ [12]. An FRT value of <18.5 cm was set as the criterion for risk of falls [13].

Executive function was measured using a reaction time measuring device and the Stroop task. The Stroop interference time was calculated and used as an executive function index, as described previously [14]. The Stroop task was also used to determine the correct task response rate of the participants. The reaction time and task correct response rate for the Stroop task were recorded after two practice sessions. The time required for the Timed Up and Go (TUG) test, which was used as an indicator of the mobility of the participants, was measured using a multi-timer (Takei Kiki Kogyo Co., Ltd., Tokyo, Japan).

**Figure 1:**
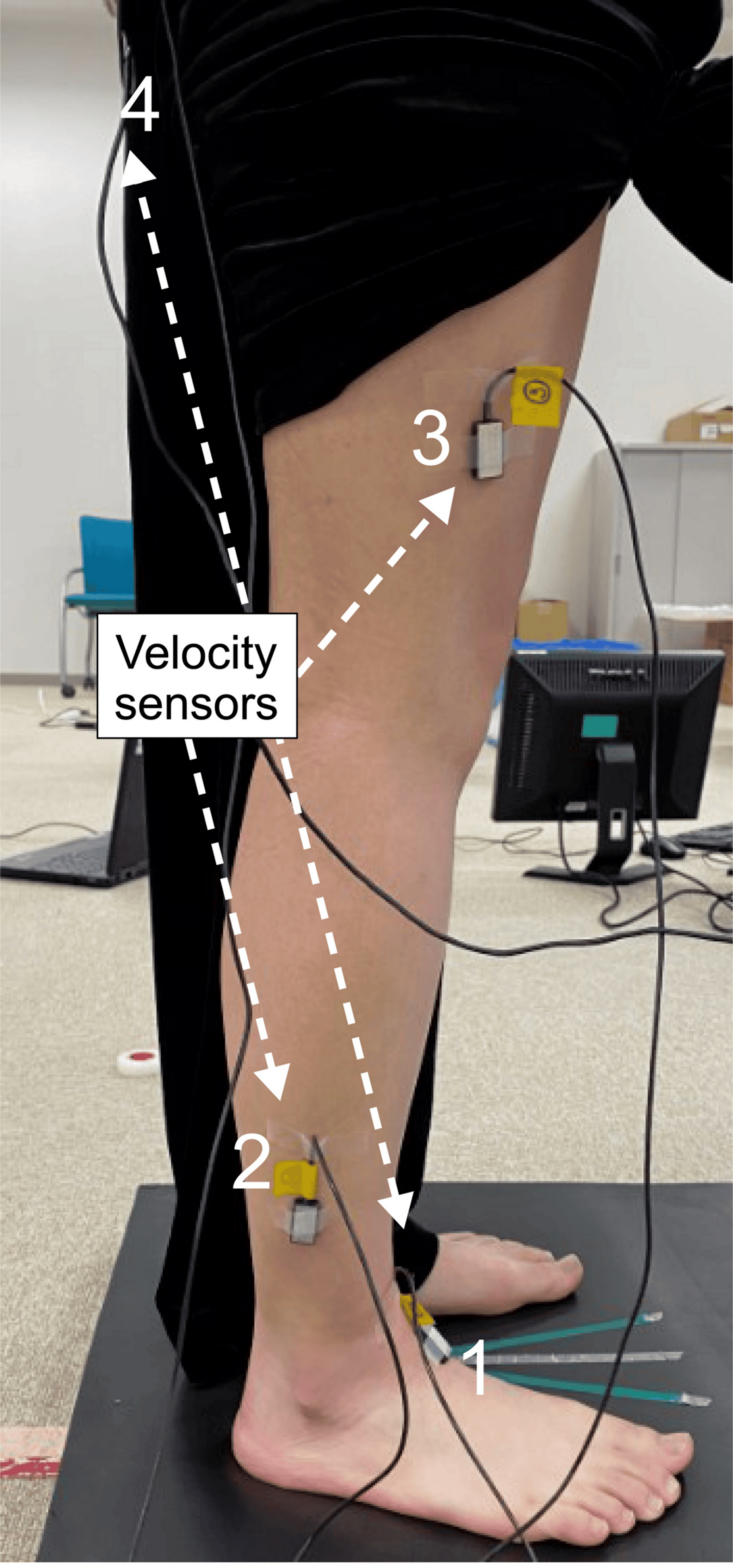
Measurement of cumulative joint angles using angular velocity sensors. The angular rate sensors were attached to four body-part areas: 1 (foot), 2 (lower leg), 3 (thigh), and 4 (back of the pelvis). The cumulative angles of the ankle joint were measured based on sensors 1 and 2, those of the knee joint based on sensors 2 and 3, and those of the hip joint based on sensors 3 and 4.

The participants were instructed to assume a stationary upright position under three conditions: stable, unstable, and unstable with executive function tasks. The postural control in these settings was quantitatively assessed using a center-of-gravity sway meter and angular velocity sensors. Triaxial angular velocity sensors (MicroStone Co., Ltd., Tokyo, Japan) were attached to the dominant foot, lower leg, thigh, and sacral region of the participants, as described in previous studies (Figure 1) [15]. The angular velocities during the task were recorded at a sampling frequency of 200 Hz, and the collected data were analyzed using a laptop equipped with a program for converting angular velocity to cumulative angle. The cumulative angles of the ankle joints were calculated using the angular velocities of the foot and lower leg. The cumulative angles of the knee joint were computed from the angular velocities of the lower leg and thigh. The cumulative angles of the hip joint were computed from the angular velocities of the thigh and sacral region. All cumulative angles were assumed to be in motion in the sagittal plane. The participants were instructed with bare feet on a center-of-gravity sway meter (GP-7; Anima Co., Ltd, Tokyo, Japan) after attaching the angular velocity sensor. The participants were subsequently instructed to perform a screen task 2 m ahead, and the total trajectory length was measured. The distance between the set was maintained at 10 cm while acquiring the measurements owing to risk of falls among older adults. The participants performed the following three-minute trials under the three conditions: (i) Stationary standing with gaze at the forward star; (ii) stationary standing with gaze at the forward star on an unstable surface (foam pad); and (iii) Stroop task + stationary standing on an unstable surface. A 10-minute rest interval was provided between the three measurements. The participants were unaware of which of the balance or tasks was given priority. The center-of-gravity sway meter or angular velocity data were obtained one to two minutes after commencing the task. The angular velocity data were converted to joint angles by moving the average and integral processing. The cumulative angle of the joint motion during the task was calculated. All cumulative angle measurements of joint motion during the task were performed by one examiner, and the intra-assessor reliability was calculated.

Data analyses

The study participants were categorized into the following three groups based on age, SMI, and FRT cut-off values: the young adult, older adult, and at-risk older adult (which comprised individuals at risk of developing reduced skeletal muscle mass and FRT of <18.5 cm) groups. The Kruskal-Wallis test and Dan Bonferroni’s multiple comparison tests were used to compare basic attributes, total trajectory length during the task, and cumulative angles at the ankle, knee, and hip joints among the three groups. Spearman’s rank correlation coefficient tests were used to examine the correlations of the cumulative angles of joint motion in the three measurement conditions with the FRT, TUG, and executive function indices. Reproducibility of the cumulative angle of joint motion was determined using intraclass correlation coefficient (ICC). ICC values ≥ 0.90 were excellent, 0.70-0.89 were considered good, 0.40-0.69 were acceptable, and < 0.40 were low. ICC (1.1) was obtained from the values calculated from the first and second measurements of the cumulative angles at the ankle, knee, and hip joints. Statistical analyses were conducted using SPSS Statistics 28 (IBM Corp., Armonk, NY, USA). The level of significance was set at 5%.

## Results

A total of 21, 25, and 12 participants were included in the young adult, older adult, and at-risk older adult groups, respectively. The at-risk older adult group was characterized by significantly lower SMI (χ^2^ = -19.89; p = .003, young adult group > at-risk older adult group, χ^2^ = -15.19; p < 0.031, older adult group > at-risk older adult group) and FRT values (χ^2^ = -40.01; p < 0.001, young adult group > at-risk older adult group, χ^2^ = -19.56; p = 0.003, older adult group > at-risk older adult group) and significantly higher Stroop interference time (χ^2^ = 29.16; p < 0.001, young adult group < at-risk older adult group, χ^2^ = 14.76; p = 0.038, older adult group < at-risk older adult group) than the other two groups. No significant differences in age, BMI, TUG time, and correct Stroop task response rates were observed between the older adult and at-risk older adult groups (Table 1).

**Table 1:** Physical and cognitive characteristics of the participants. Values are median and 25th–75th percentile. The P-value (inter-group) was calculated by the Kruskal-Wallis H test, and the P-value (intra-group) was calculated by Dan Bonferroni’s multiple comparison tests. Group A: young adult group, Group B: older adult group, Group C: at-risk older adult group, BMI: body mass index, SMI: skeletal muscle mass index, FRT: functional reach test, TUG: timed up and go test, SI time: Stroop interference time, ST percentage correct: Stroop test percentage correct.

Characteristics	Group A (n=21)	Group B (n=25)	Group C (n=12)	H-test statistic	p	p (post hoc) A-B A-C B-C		
									Age (years)	21.0 (21.0-21.0)	71.0 (69.0-73.0)	78.0 (71.5-79.8)	43.1	< 0.001	< 0.001	< 0.001	0.278
BMI (kg/m^2^)	20.1 (19.3-21.8)	23.9 (20.9-26.4)	22.7 (20.8-29.4)	10.0	0.007	0.011	0.050	0.990
SMI (kg/m^2^)	6.1 (5.6-6.6)	5.9 (5.5-6.5)	5.4 (5.2-5.6)	11.0	0.004	0.346	0.003	0.031
FRT (cm)	35.4 (33.0-38.1)	26.0 (21.5-29.8)	15.3 (11.1-17.5)	44.7	< 0.001	< 0.001	< 0.001	0.003
TUG (sec)	6.4 (5.9-6.9)	7.1 (6.4-8.0)	9.0 (7.7-9.7)	21.5	< 0.001	0.024	< 0.001	0.067
SI time (msec)	119.1 (20.1-190.7)	258.8 (171.6-331.4)	638.7 (259.9-852.8)	23.5	< 0.001	0.012	< 0.001	0.038
ST percentage correct (%)	93.3 (86.7-96.7)	93.3 (86.7-96.7)	85.0 (74.2-92.3)	12.6	0.002	0.111	0.001	0.191

Evaluation of the standing postural control revealed significant differences in the total trajectory length between the young adult and older adult groups and between the young adult and at-risk older adult groups in the three measurement conditions; however, no significant differences were observed between the older adult and at-risk older adult groups (Tables 2-4). Cumulative angles of lower limb joint motion in the static standing position varied among the groups in terms of the ankle joint under measurement conditions (1) and (2) (Tables 2, 3). Intergroup variations were observed in terms of the ankle, knee, and hip joints under executive function tasks and unstable floor conditions; however, the cumulative angle of the hip joint significantly increased in the at-risk older adult group (Table 4). The ICC (1, 1) for the cumulative angle measurement of joint movements during the task for the ankle, knee, and hip joints were 0.86, 0.85, and 0.74, respectively. Negative correlations were observed between the cumulative knee angle and FRT values (r = -0.450, p < .001) and between the cumulative hip angle and FRT values (r = -0.641, p < .001), whereas a positive correlation was observed between the cumulative hip angle and Stroop interference time (r = 0.347, p = 0.008) under the measurement condition (3) (Table 5).

**Table 2:** Cumulative angles of joint motion and center of pressure (COP) while the standing position on the floor. Values are median and 25th–75th percentile. The P-value (inter-group) was calculated by the Kruskal-Wallis H test, and the P-value (intra-group) was calculated by Dan Bonferroni’s multiple comparison tests. Group A: young adult group, Group B: older adult group, Group C: at-risk older adult group, LNG: the total length of the sway of the center of pressure.

	Group A (n=21)	Group B (n=25)	Group C (n=12)	H-test statistic	p	p (post hoc) A-B A-C B-C		
									Ankle (degree)	7.7 (6.3-8.7)	8.9 (7.8-10.5)	8.9 (7.8-10.5)	9.7	0.008	0.042	0.016	1.000
Knee (degree)	9.4 (7.6-13.9)	11.0 (9.5-12.3)	10.4 (7.2-12.8)	1.2	0.540			
Hip (degree)	17.4 (12.1-20.4)	18.5 (15.2-20.8)	19.0 (13.8-27.3)	0.6	0.746			
LNG (cm)	42.7 (39.1-51.9)	65.1 (53.8-77.8)	64.7 (52.0-80.8)	22.2	< 0.001	< 0.001	< 0.001	1.000

**Table 3:** Cumulative angles of joint motion and center of pressure (COP) while the standing position on the unstable plate. Values are median and 25th–75th percentile. The P-value (inter-group) was calculated by the Kruskal-Wallis H test, and the P-value (intra-group) was calculated by Dan Bonferroni’s multiple comparison tests. Group A: young adult group, Group B: older adult group, Group C: at-risk older adult group, LNG: the total length of the sway of the center of pressure.

	Group A (n=21)	Group B (n=25)	Group C (n=12)	H-test statistic	p	p (post hoc) A-B A-C B-C		
									Ankle (degree)	12.7 (11.5-18.4)	17.6 (14.9-31.1)	21.2 (17.5-27.5)	17.2	0.002	< 0.001	0.019	0.864
Knee (degree)	13.9 (10.0-17.9)	15.6 (12.8-21.4)	18.5 (15.6-21.6)	4.0	0.133			
Hip (degree)	19.6 (16.6-24.9)	23.0 (18.3-30.5)	24.5 (21.7-29.1)	4.2	0.125			
LNG (cm)	63.4 (59.3-80.2)	94.6 (82.2-125.0)	91.2 (85.5-109.8)	30.3	< 0.001	< 0.001	< 0.001	1.000

**Table 4:** Cumulative angles of joint motion and center of pressure (COP) while standing under Stroop task and unstable plate conditions. Values are median and 25th–75th percentile. The P-value (inter-group) was calculated by the Kruskal-Wallis H test, and the P-value (intra-group) was calculated by Dan Bonferroni’s multiple comparison tests. Group A: young adult group, Group B: older adult group, Group C: at-risk older adult group, LNG: the total length of the sway of the center of pressure.

	Group A (n=21)	Group B (n=25)	Group C (n=12)	H-test statistic	p	p (post hoc) A-B A-C B-C		
									Ankle (degree)	17.1 (12.0-23.0)	20.6 (18.7-28.4)	25.5 (19.7-30.6)	7.3	0.026	0.104	0.044	1.000
Knee (degree)	13.1 (10.8-20.6)	19.7 (16.6-26.9)	25.5 (19.1-30.9)	13.3	0.001	0.030	0.002	0.493
Hip (degree)	18.5 (14.7-20.7)	24.1 (18.7-30.5)	35.1 (28.1-41.3)	21.4	< 0.001	0.022	< 0.001	0.043
LNG (cm)	62.7 (48.8-85.9)	106.6 (95.2-129.4)	107.7 (84.8-145.5)	32.5	< 0.001	< 0.001	< 0.001	1.000

**Table 5:** Correlations between the assessment items and cumulative angles of joint motion while standing under Stroop task and unstable plate conditions. TUG: timed up and go, SI time: Stroop interference time, FRT: functional reach test, *: p < 0.05.

	TUG	SI time	Cumulative angles of ankle joint	Cumulative angles of knee joint	Cumulative angles of hip joint
TUG		0.517*	0.219	0.239	0.229
SI time	0.517*		0.160	0.238	0.347*
FRT	- 0.595*	- 0.620*	- 0.226	- 0.450*	- 0.644*

## Discussion

This study examined differences in standing postural control strategies to maintain posture on floor surfaces of varying stability among young adults, older adults, and at-risk older adults using cumulative angles. To the best of our knowledge, this study is the first to investigate the impact of cognitive demands on standing postural control, utilizing both executive and non-executive function tasks on unstable floor surfaces.

Among the female participants included in the three groups in this study, those included in the older adult and at-risk older adult groups had significantly higher BMI and significantly lower SMI. Lower skeletal muscle density indicates greater fat infiltration into muscle, which is associated with increased risk of fracture in older adults [16]. At-risk older adults had an SMI of 5.4 kg/m^2^ in the present study, indicating reduced skeletal muscle mass, consistent with previous findings, indicating that individuals with skeletal muscle loss are at increased risk for falls [17].

The present study focused on standing postural control; however, the reaction time of a computerized executive function task was also measured. Executive function was measured similarly to that in previous functional neuroimaging studies, wherein the Stroop interference times were calculated [14]. Activation of the lateral prefrontal cortex in both hemispheres has been reported during executive function tasks [14]. As reported previously, executive function was affected by aging. Functional brain connectivity during tasks is stronger in younger participants. The reduced functional connectivity in older individuals is attributed to white matter atrophy, which reduces the neural activity in brain regions directly related to executive function tasks [18]. Furthermore, the decline in executive function is stronger in at-risk older individuals than in the other two groups. This finding supports the relationship between participants with poor executive function, poorer reaction times and postural sway, and the risk of multiple falls [19].

In the present study, angular velocity sensors were used to calculate the cumulative angles of each lower limb joint during the task, as described previously [15]. In recent years, several motion analysis systems based on wearable inertial sensors have been developed. These systems offer a promising replacement for standard motion capture systems for the measurement of gait and joint kinematics parameters with high reliability and excellent validity. Angular velocity sensors can assess the static equilibrium ability of preschool-aged children and are highly reliable [20]. The cumulative angles of the ankle, knee, and hip joints were calculated using angular velocity sensors. The reliability ICC (1, 1) of the cumulative angle measurement during the task was high for the ankle (0.86), knee (0.85), and hip (0.74) joints.

Standing postural control is more strongly influenced by ground stability in older adults than in younger adults, which may be attributed to the muscle mass required for postural control decreasing with age [21]. Less active older adults are particularly prone to balance problems [21], and reduction in SMI and FRT values among older adults may corroborate these findings. The results of the present study indicate that increased ankle joint mobility in older adults compensates for postural control on unstable floor surfaces. The cumulative angles of the knee and hip joints were increased in all groups; however, this was not due to aging (Tables 2, 3). Relationships between increased COP, physiological tremors, and decreased muscle mass in the lower limb during stationary standing in older individuals [22], as well as increased postural sway with increased task difficulty [23], have been observed. Older female participants show increased COP velocities during postural adjustment [23], which may contribute to increased cumulative ankle joint angles on unstable floor surfaces among older adults and at-risk older adults compared with those among young adults. However, the settings, including task difficulty and COP measurement conditions, should be clarified in future studies.

Older adults exhibit stronger postural sway than younger adults even without cognitive demands or use of executive function tasks, which may impose lower-order cognitive demands owing to task difficulty, resulting in greater sway [24]. However, CMDT-based research continues owing to the age-related reduction in executive function resources required to coordinate cognitive and postural demands. Older adults and at-risk older adults may experience difficulty in cognitive-motor dual-task situations that require executive function. A significant increase in the COP total length was observed in older adults and at-risk older adults compared with that in younger adults in situations using executive function tasks and under unstable floor conditions. In addition, a significant increase in cumulative knee and hip joint angles was observed. Previous CMDT studies examined postural control while performing dual-task as the change in COP and postural sway [25]. An increase in COP parameters is not necessarily linked to postural instability, although it may reflect a stable postural control that makes continuous postural adjustments to stabilize the center of mass, provided that the COP does not exceed the basal plane of support [26]. Postural control mechanisms use the ankle joint strategy when the posture is undisturbed and slow or small-amplitude perturbations occur. The hip joint strategy is used in conditions wherein rapid or great-amplitude perturbations occur [27]. The gradual loss of ankle-stabilizing muscle strength in the aged leads to delayed activation of the trunk and thigh muscles and reliance on the hip strategy, especially when a stable upright posture is threatened. Older adults tend to depend on hip strategies more often than younger adults, which has not been adequately observed in COP measurements [28]. Older individuals cannot maintain standing balance with the ankle joint alone [26], indicating more antagonist muscle activation and use of a hip strategy. When performing executive function task, postural control shows a significant increase in the cumulative hip angle in older adults and at-risk older adults and a significant increase in hip angle in older adults. Mixed hip and ankle joint activation increases postural sway. The cumulative hip angle is increased in at-risk older adults compared with that in older adults, which may contribute to risk of falls. Relationships between proximal femoral fractures, falls, and reduced muscle mass have been reported in aging research [29]. The activation of hip strategies for postural control may develop into risk of falls in at-risk older adults owing to reduced SMI; however, this hypothesis remains to be verified.

The limitations of this study include differences in the ethnicity of the participants and the small sample size. The calculated sample size was not achieved due to COVID-19 restrictions. Therefore, the findings of this study should be interpreted with caution. Furthermore, in addition to COP, the cumulative angles of the lower limb joints were measured to determine postural instability. Hip strategies play an important role in maintaining postural control in older individuals, especially lateral control [30]. Future studies should examine the use of executive function tasks for postural and lateral control of the hip joints in older individuals. Despite these limitations, our study design had the advantage of enabling us to identify postural sway as well as sway-related increases in lower extremity joint motion for standing postural adjustments. Difficulty of the task and use of this measurement system can aid in screening for risk of falls among older adults.

## Conclusions

This study highlights the intricate relationship between aging, cognitive function, and postural control, particularly in the context of fall risk among older adults. The cumulative angles of the hip and knee joints were increased in older adults and at-risk older adults when an executive function task was performed on an unstable surface. These findings provide novel insights into the reduced cognitive work required to compensate for postural regulation and increased instability and activation of hip strategies for postural maintenance via age-related deficits in executive function. The risk of falls is more severe with the use of hip strategies, particularly among at-risk older adults with reduced muscle mass. By examining standing postural control strategies across different age groups and cognitive demands, the study provides valuable insights into the mechanisms underlying postural instability and its implications for fall prevention.
